# Insight into higher-level phylogeny of Neuropterida: Evidence from secondary structures of mitochondrial rRNA genes and mitogenomic data

**DOI:** 10.1371/journal.pone.0191826

**Published:** 2018-01-30

**Authors:** Nan Song, Aili Lin, Xincheng Zhao

**Affiliations:** College of Plant Protection, Henan Agricultural University, Zhengzhou, China; Sichuan University, CHINA

## Abstract

It is well known that the rRNA structure information is important to assist phylogenetic analysis through identifying homologous positions to improve alignment accuracy. In addition, the secondary structure of some conserved motifs is highly stable among distantly related taxa, which can provide potentially informative characters for estimating phylogeny. In this paper, we applied the high-throughput pooled sequencing approach to the determination of neuropteran mitogenomes. Four complete mitogenome sequences were obtained: *Micromus angulatus* (Hemerobiidae), *Chrysoperla nipponensis* (Chrysopidae), *Rapisma* sp. (Ithonidae), and *Thaumatosmylus* sp. (Osmylidae). This allowed us to sample more complete mitochondrial RNA gene sequences. Secondary structure diagrams for the complete mitochondrial small and large ribosomal subunit RNA genes of eleven neuropterid species were predicted. Comparative analysis of the secondary structures indicated a closer relationship of Megaloptera and Neuroptera. This result was congruent with the resulting phylogeny inferred from sequence alignments of all 37 mitochondrial genes, namely the hypothesis of (Raphidioptera + (Megaloptera + Neuroptera)).

## Introduction

Neuropterida is a name sometimes applied to an insect superorder within Holometabola. This superorder comprises three orders: Raphidioptera (snakeflies), Megaloptera (dobsonflies) and Neuroptera (lacewings). Some insect species of Neuropterida are economically important, owing to their significant role played by adults and/or larvae in the bio-control of insect pest species on agricultural crops [1,2]. Moreover, some species of Neuropterida exhibit an exceptionally wide range of morphological and biological diversity. Approximately 200 described species have been placed in two families of Raphidioptera, about 300 species in two families of Megaloptera, and about 5,800 species in 15 or 16 families of Neuroptera [3]. Furthermore, it is estimated that there are 10,000 extant neuropteridan insect species in the world [4–6]. Therefore, the Neuropterida are recognized as the fifth largest assemblage among the holometabolan groups [7].

Although with the economic importance and relatively rich fossil evidence, the phylogenetic position of the entire Neuropterida and interrelationships among the three orders of Neuropterida have been the most contentious questions in the higher-level phylogenetics of Holometabola. The Neuropterida is commonly considered to be one of the most basal lineage within Holometabola [6]. The Coleoptera is typically identified to be the sister group of Neuropterida [7–10]. However, Boudreaux (1979) [11] suggested the Mecopterida (including Mecoptera, Siphonaptera, Diptera, Trichoptera and Lepidoptera) to be the sister group of Neuropterida. Alternatively, the groupings of (Mecoptera + Siphonaptera) [12], ((Lepidoptera + Trichoptera) + (Mecoptera + Siphonaptera)) [13], (Strepsiptera + (Coleoptera + Diptera)) [14], and (Strepsiptera + Coleoptera) [15] were respectively proposed as the sister group of Neuropterida. More recently, a genome-scale study recovered the clade (Strepsiptera + Coleoptera) as the sister group of Neuropterida [16]. In our prior analyses of the entire Holometabola phylogeny based on mitogenomic data, the sister group relation between the Neuropterida and the clade (Strepsiptera + Coleoptera) was the mostly recovered relationship [17]. Another recent mitogenomic study also supported the same sister group relationship of Neuropterida and the clade (Strepsiptera + Coleoptera) [18]. In short, the majority of recent studies tended to reveal a close affinity of Neuropterida with Coleoptera.

In contrast to the unstable phylogenetic position of Neuropterida, the monophylies of the entire Neuropterida and of both Raphidioptera and Neuroptera have been generally accepted. Morphological evidence supporting the monophyly of Neuropterida have been summarized by Aspöck et al. (2012) [19]. Furthermore, the monophyletic Neuropterida was confirmed by several molecular studies [4,16–18]. Recently, the monophyly of Neuropterida was supported by a phylogenomic study based on anchored hybrid enrichment data [20]. Some earlier studies indicated Megaloptera as a non-monophyletic assemblage [15,21,22]. However, much more researches supported Megaloptera to be monophyletic [4,16–18,20,23–25]. For the inter-ordinal relationships within Neuropterida, three possible arrangements have been proposed. Traditionally, the sister group of (Megaloptera + Raphidioptera) was hypothesized in the context of morphological analyses [8,9,21]. Song et al. (2016) recovered a clade of (Neuroptera + (Megaloptera + Raphidioptera)) based on the mitogenomic data [18]. This relationship was also recovered by *18S rRNA* data [26]. Wheeler et al. (2001) [27] provided an unresolved trichotomy of the three orders within Neuropterida based on the combined analysis of nuclear *18S rRNA* and *28S rRNA* gene sequences. In contrast, several authors provided good support for the sister-group relationship between Megaloptera and Neuroptera based on complete mitogenome data [7,28] and genome-wide data [16,20]. This relation was corroborated by morphological data of genital sclerites [23] and wing base structures [29].

As mentioned above, complete mitogenomes have been used to infer Neuropterida relationships at different phylogenetic levels [7,17,18,25,28,30], with various degree of success. However, these studies were based on the traditional Sanger sequencing, by which reconstructing mitogenomes were time and cost consuming due to the difficulty of long PCR and the necessity of designing a large set of species-specific PCR primers for primer-walking [31]. The advent of next-generation sequencing (NGS) technologies is revolutionizing the current biology. Specifically, NGS approaches have made insect mitogenome reconstruction to be less labor-intensive and more cost-effective. Several recent studies have successfully employed the NGS methods to assemble mitogenome sequences, which allow for multiplexing samples from diverse taxa and maximizing the number of taxa involved in the research [32–36].

The relative ease of acquiring complete mitogenomes by NGS methods further contributes to the mitogenomic phylogeny researches. Of the 37 mitochondrial genes, the mitochondrial small (*rrnS*) and large (*rrnL*) ribosomal subunit RNA sequences have been the most widely used phylogenetic markers [37–45]. The total length of both *rrnS* and *rrnL* gene sequences of neuropterid species is approximately 2.1 kb, which makes up *ca*. 14% phylogenetic information of the whole mitogenome. However, explicitly aligning these mitochondrial ribosomal RNA sequences is generally difficult, because of the variable sequence length and the indel-rich regions contained in both gene sequences. Inexactly aligned nucleotide sequences do not correctly reflect homology between sequences [46–48], which might lead to artificial phylogenetic hypotheses. Several previous studies have shown that the secondary reconstruction of ribosomal *RNA* molecules can aid in improving recognition of primary homology [46,51,52], and refine the alignment process [46–50]. Some structural motifs are highly stable among distantly related taxa, which can provide potentially informative characters for estimating phylogeny [53].

In the present study, we applied high-throughput pooled sequencing approach to reconstruct four complete mitogenomes of Neuroptera, and used them to investigate the interrelationships among Raphidioptera, Megaloptera and Neuroptera. In addition, secondary structures of the complete mitochondrial *rrnS* and *rrnL* genes were presented for eleven neuropterid species to provide new evidence for the phylogeny of Neuropterida.

## Materials and methods

### Ethics statement

No specific permits were required for the insect specimens collected for this study in China. The specimens of *Micromus angulatus*, *Chrysoperla nipponensis* EMHAU-15090613, and *Thaumatosmylus* sp. were collected by authors in Xinyang, Henan, China, while the *Rapisma* sp. was collected Linzhi, Tibet, China. The field studies did not involve endangered or protected species. The species sequenced in this study is not included in the ‘‘List of Protected Animals in China”.

### Taxon sampling

Tissue samples from four species of Neuroptera, including *M*. *angulatus* (Hemerobiidae), *C*. *nipponensis* EMHAU-15090613 (Chrysopidae), *Rapisma* sp. (Ithonidae) and *Thaumatosmylus* sp. (Osmylidae) were collected for DNA extraction and subsequent sequencing.

In the phylogenetic analysis, a total of 35 taxa were included for tree reconstruction. Two species of Coleoptera were selected as the outgroup taxa. Ingroups included 33 species representing three orders of Neuropterida. S1 Table lists the taxa analyzed in this study.

### De novo assembly of mitogenomes

Genomic DNA was extracted from 95% ethanol-preserved specimen individually from the thoracic leg muscle tissue using the TIANamp Micro DNA Kit (TIANGEN BIOTECH CO., LTD) as per the manufacturer’s protocol. DNA concentration was measured by Nucleic acid protein analyzer (QUAWELL TECHNOLOGY INC.).

Uniform quantities of genomic DNA from each of the neuropteran insects were pooled, and DNA was concentrated to 1.5 μg. The mixed DNA sample was utilized for library construction, using Illumina TruSeqTM DNA Sample Prep Kit (Illumina, San Diego, CA, USA) and with an average insert size of 350 bp. The subsequent de novo genome sequencing were conducted on the Illumina HiSeq2500 platform in Shanghai OE Biotech CO., LTD. For the sequenced sample, 10 Gb paired-end reads of 125 bp length were generated. FastQC [54] was used for the quality control checks on raw sequence data. NGS QC toolkit [55] was applied to filter the data (cutoff read length for HQ = 70%, cutoff quality score = 20). In this step, reads containing adapters and ploy-N, and low quality reads were removed from raw data. At the same time, Q20, Q30, GC-content and sequence duplication level of the cleaned data were calculated. All the downstream analyses were based on clean data with high quality (avg. Q20 > 90%, and avg. Q30 > 85%). Finally, no less than 8 Gb high-quality reads were used in de novo assembly with IDBA-UD v. 1.1.1 [56]. The assemblies were constructed using 200 for the setting of minimum size of contig, and an initial k-mer size of 40, an iteration size of 10, and a maximum k-mer size of 90.

To identify the mitogenome assemblies from the pooled sequencing files, three different fragments of mtDNA (*cox1* 5’ region, *cytb* 3’ region, and *rrnS* 5’ region) were amplified as “Bait” sequences by standard PCR reactions using universal primers designed by the study of Song et al. (2016) [57]. Local BLAST searches were conducted with BioEdit version 7.0.5.3 [58] for each bait sequence reference against all corresponding assemblies. Only hit with 100% pairwise identity was considered as a successful identification. The mitochondrial contigs identified were inputted into MITOS [59] for initial mitogenome annotation. The resultant gene boundaries were checked and corrected by alignment with published Neuroptera mitogenome sequences (see the detailed species names in S1 Table). Additionally, we conducted mappings for the identified mitochondrial contigs using BWA v 0.7.13 [60], under default parameters. Mapping statistics were obtained to check the quality of the assembling with Qualimap [61] and Tablet [62], respectively.

### Sequence alignment

All thirty-seven genes of insect mitogenome, including 13 protein-coding genes, 22 *tRNA* genes and two *rRNA* genes, were respectively aligned and used to further analyses. For protein-coding genes, firstly stop codons were excluded. Subsequently, each was aligned based on the invertebrate mitochondrial genetic code with Perl script TransAlign [63]. Each of *tRNA* and *rRNA* genes was aligned using MAFFT (version 7) under iterative refinement method incorporating the most accurate local (E-INS-i) pairwise alignment information [64]. The resultant alignments were checked in MEGA 6 [65]. Gaps were automatically striped by Gap Strip/Squeeze v2.1.0 with 40% Gap tolerance (http://www.hiv.lanl.gov/content/sequence/GAPSTREEZE/gap.html). All alignments were concatenated into the final data matrices with FASconCAT_v1.0 [66]. Two classes of data were compiled, namely with or without RNA genes.

To reduce the random similarity in the sequence alignments, each alignment of protein-coding genes, *tRNA* genes and *rRNA* genes was individually masked with Aliscore version 2.0 [67,68]. The sequence alignments were screened separately with the following options: -r 2000, -w 6, -N, -o outgroups. Positions identified as randomly similar by Aliscore were removed using Alicut version 2.0 [67,68]. After masking, the single gene alignments were concatenated with FASconCAT.

Potential saturation in the combined data sets was assessed using the index of substitution saturation (*Iss*) implemented in the DAMBE 5 [69,70]. Nucleotide homogeneity across taxa was assessed using the Chi-square test [71] implemented in PAUP*4.0b10 [72]. In addition, the results of IQ-TREE [73,74] also contain the Chi-square test, by which we can compare the composition of each sequence to the average composition across all data. The settings of IQ-TREE were the same as the following section of phylogenetic analysis.

Estimates of nonsynonymous (*dN*) and synonymous (*dS*) substitution rates of concatenated protein-coding genes were obtained by Yang and Nielsen (2000) [75] method using the program yn00 as implemented in PAML 4.9 [76]. The One-way Analysis of Variance (ANOVA) is performed in Excel 2016. In addition, the evolutionary rates of individual protein-coding genes were calculated using the same method mentioned above. Divergences of alignments of each protein-coding gene, *rrnL*, *rrnL*-SS, *rrnS*, *rrnS*-SS and *tRNA* were calculated using MEGA with the Maximum Composite Likelihood model.

### Secondary structure prediction

**Fig 1:**
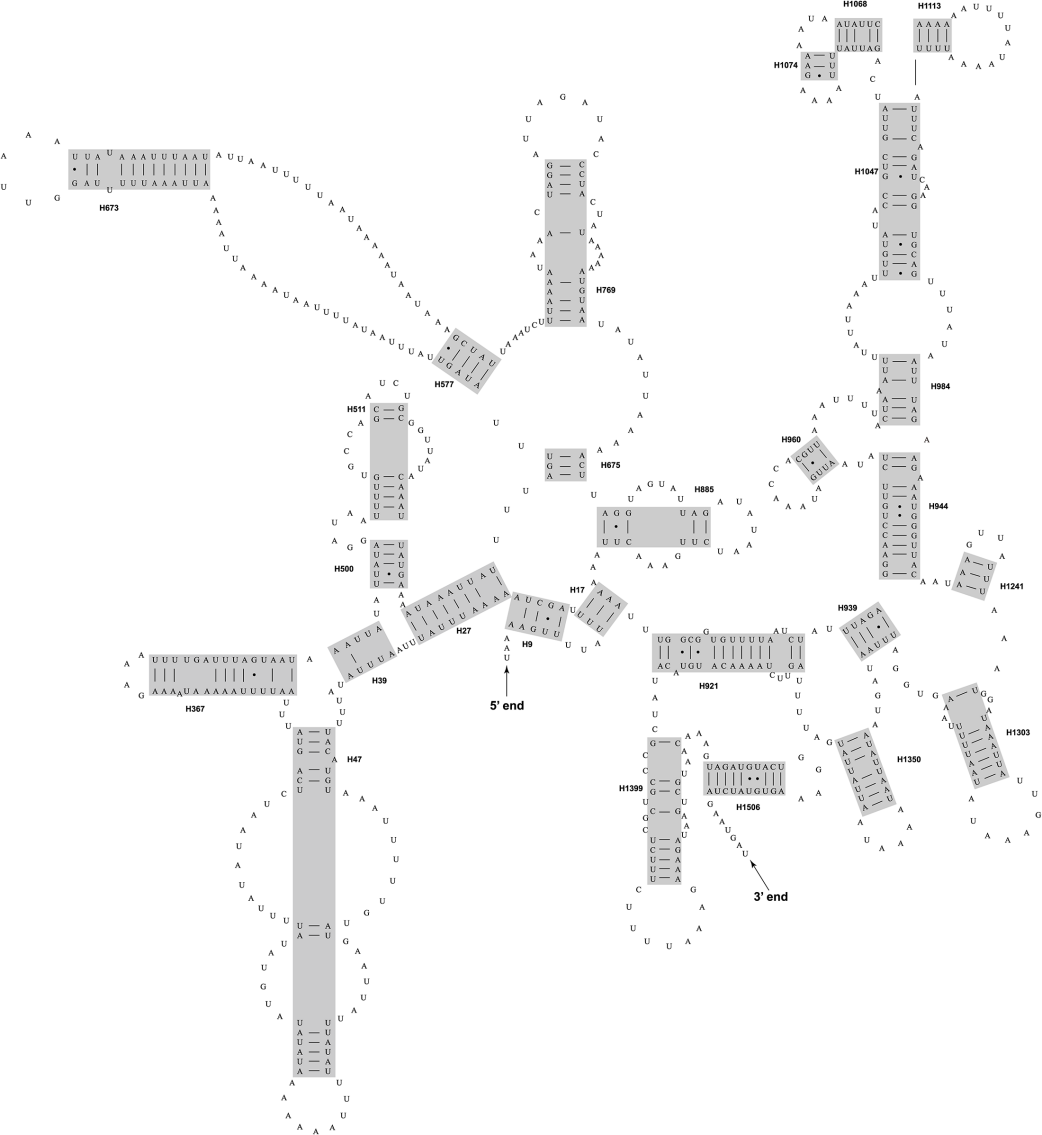
Secondary structure drawing of the complete mitochondrial *rrnS* gene for *Chrysoperla nipponensis* EMHAU-15090613. The grey boxes indicated the helices aligned across all eleven sampled neuropterids.

**Fig 2:**
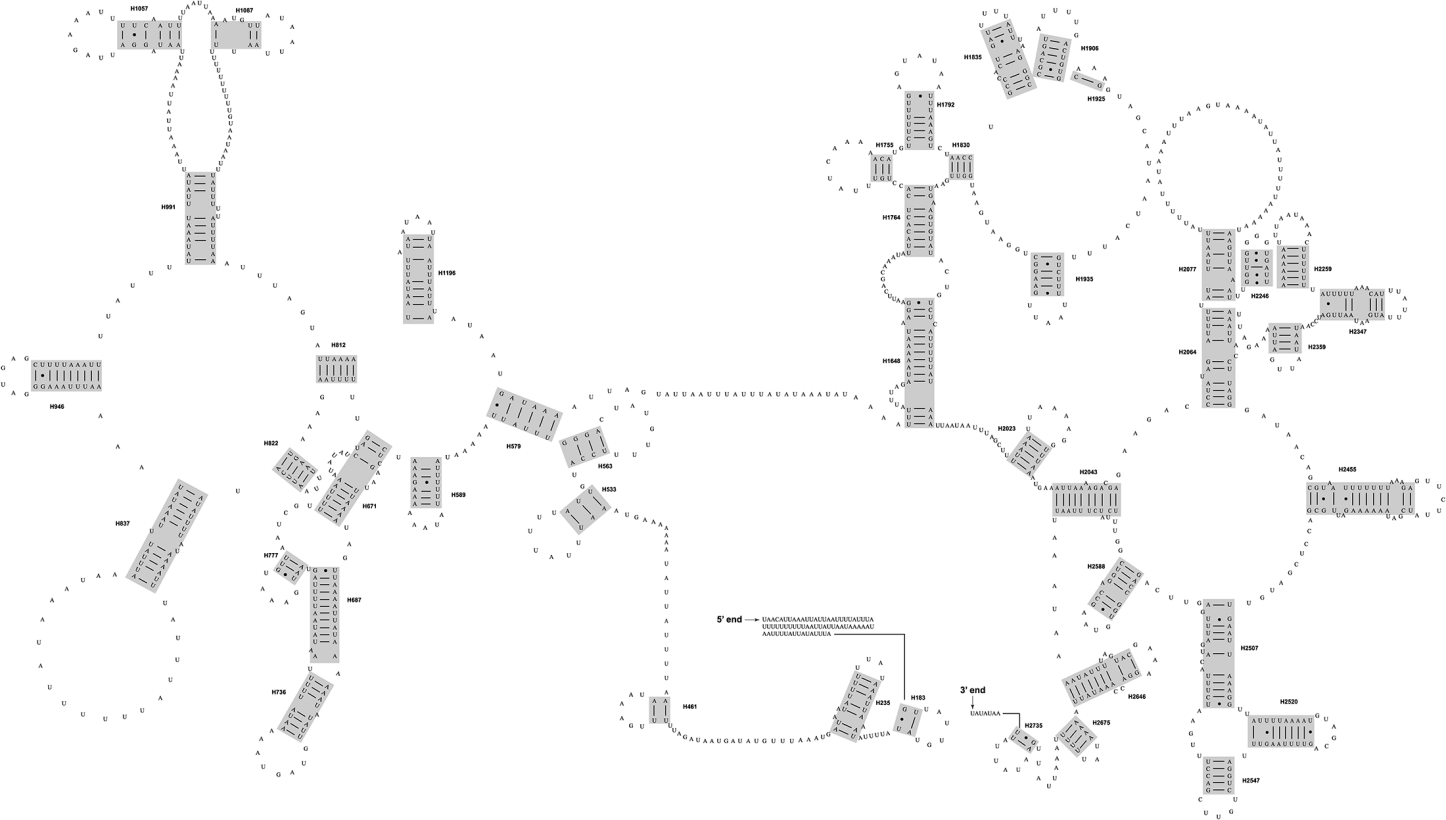
Secondary structure drawing of the complete mitochondrial *rrnL* gene for *Chrysoperla nipponensis* EMHAU-15090613. The grey boxes indicated the helices aligned across all eleven sampled neuropterids.

The secondary structures of mitochondrial *rrnS* and *rrnL* were predicted mainly with reference to the method of “Comparative sequence analysis” by Ouvrard et al. (2000) [52]. Slight modifications were implemented in this study. Firstly, we used the MAFFT under the method of E-INS-i to produce the preliminary alignments, on which we could identify the conserved regions shared by the analyzed taxa and distinguish the hyper-variable portions present in each individual species. In the second step, the secondary structures of each conserved region and of each hyper-variable region were inferred, with reference to the models predicted for *Apis mellifera* [77] and/or *Drosophila virilis* [78]. Thirdly, putative helices were located through searching for uninterrupted base pairing in regions similar to the model of *A*. *mellifera* [77] or *D*. *virilis* [78]. Finally, Helix numbering followed the convention established at the CRW site [78]. The secondary structure diagrams of the complete mitochondrial *rrnS* and *rrnL* for eleven neuropterid insects are presented in Figs 1 and 2, Figures A-J in S1 Fig and Figures A-J in S2 Fig.

Based on secondary structures, sequences of *rrnS* and *rrnL* were realigned. Furthermore, both alignments were concatenated with subsets of protein-coding genes and *tRNA* genes, in order to investigate the effect of different sequence refinement algorithms on the phylogenetic reconstructions.

### Phylogenetic analysis

#### Data partitioning

Prior to phylogenetic analyses, PartitionFinder [79] was employed to infer the optimal partitioning strategy, under a greedy search with RAxML [80]. For the data sets of PCG and PCGRNA, the data blocks were defined by gene types and by codon positions. For the remaining data sets, the data blocks were defined only by gene types. The partition schemes for each data set are provided in Tables A-N of S2 Table.

#### Phylogenetic reconstruction

The phylogenetic analyses were conducted using the following data sets: 1) PCG: 13 protein-coding genes; 2) PCG12: 13 protein-coding genes without the third codon positions; 3) PCG_AA: deduced amino acids of 13 protein-coding genes; 4) PCGRNA: combination of PCG, 2 *rRNA* and 22 *tRNA* genes; 5) PCG12RNA: combination of PCG12, 2 *rRNA* and 22 *tRNA* genes. Additionally, five corresponding data sets with masking were compiled: 1) Aliscore_PCG; 2) Aliscore_PCG12; 3) Aliscore_PCG_AA; 4) Aliscore_PCGRNA; 5) Aliscore_PCG12RNA.

To investigate the effect of mitochondrial *rRNA* realignments derived from secondary structures, eight corresponding data sets were utilized in phylogenetic analyses: 1) *rrnS*: *rrnS* aligned automatically by MAFFT; 2) *rrnL*: *rrnL* aligned automatically by MAFFT; 3) *rrnS-*SS: *rrnS* aligned manually based on secondary structures; 4) *rrnL-*SS: *rrnL* aligned manually based on secondary structures; 5) PCGRNA-SS: combining PCG, *tRNA*, and *rRNA* sequences realigned on secondary structures; 6) PCG12RNA-SS: combining PCG12, *tRNA*, and *rRNA* sequences realigned on secondary structures; 7) Aliscore_PCGRNA-SS: PCGRNA-SS with masking; 8) Aliscore_PCG12RNA-SS: PCG12RNA-SS with masking. In total, eighteen data sets were utilized in tree searches.

Maximum likelihood (ML) analyses were conducted using IQ-TREE [73,74], with various data partition schemes and the corresponding substitution models determined by PartitionFinder (Tables A-N in S2 Table). Branch support was estimated using Ultrafast bootstrap analysis with 1000 replicates.

Bayesian analyses were performed using a parallel version of PhyloBayes (pb_mpi1.5a) [81,82] as implemented on a HP server with twenty-four CPU and 320 G memory. The model CAT-GTR was used for nucleotide analyses, while the model CAT for amino acids. Two chains were run in parallel, and started from a random topology. The bpcomp program contained in the package of PhyloBayes was used to calculate the largest “maxdiff” and mean “meandiff” discrepancy observed across all bipartitions, and to yield the final consensus tree with the default options. The program tracecomp was also used to summarize the discrepancies and the effective sizes estimated for each column of the trace file. When the maximum “maxdiff” value was lower than 0.1 and minimum effective size was higher than 100, the Bayesian runs were recognized to be reached good convergence.

For the trees obtained, the bootstrap supports (BS) ≥ 75 and the posterior probabilities (PP) ≥ 0.90 were considered to be credible support values for the internal nodes. All sequence alignment files and tree files built in this article are available in Supporting Information S1–S3 Files.

## Results

### Mitogenomic assembly

Four complete neuropteran mitogenome sequences were obtained from each of single contigs. Mapping statistics showed that every site in each contig corresponded to the same base composition. Table 1 summarizes the statistics associated to the sequencing of the mitogenome assemblies. The total number of 125 bp reads ranged from 288,827,144 (*M*. *angulatus*) to 326,450,911 (*C*. *nipponensis* EMHAU-15090613). From these sequences, 0.22% to 0.27% corresponded to mitochondrial reads. The sequencing coverage ranged from 2,759× to 3,283×. Four mitogenomes had sequence lengths typical for insects, which ranged from 15,958 bp (*Rapisma* sp.) to 16,186 bp (*Thaumatosmylus* sp.). Each of them consisted of the full 37 mitochondrial genes and a putative control region. The mitogenomes showed the gene order proposed as ancestral for insects [31], except for the arrangement of *tRNA* cluster of *trnC*(gca)-*trnW*(tca)-*trnY*(gta). The variability of genome length occurred mainly in the control regions which ranged in size from 1,166 bp (*Rapisma* sp.) to 1,310 bp (*M*. *angulatus*). The organization of each mitogenome is presented in Tables A-D of S3 Table. All newly determined sequences have been deposited in GenBank (accession numbers: KX670539-KX670542).

**Table 1:** Statistics associated to the sequencing of mitogenomes using NGS-Illumina technology in four neuropteran species.

Species Name	Total number of reads	Mitochondrial reads	Percent of mitochondrial reads	Mapped bases	Mean coverage	Length
*Micromus angulatus*	288,827,144	785,218	0.27%	49,890,452	3089×	16,151
*Chrysoperla nipponensis* EMHAU-15090613	326,450,911	729,897	0.22%	50,603,335	3153×	16,047
*Thaumatosmylus* sp.	326,450,328	720,802	0.22%	44,658,218	2759×	16,186
*Rapisma* sp.	326,450,107	707,473	0.22%	52,384,940	3283×	15,958

### Sequence characteristics of data matrices

The saturation tests showed the transitions at the third codon positions to be saturated when assuming an asymmetrical topology (*Iss* > *Iss*.*cAsym*, Table 2). Nevertheless, all the remaining data partitions passed the test (*Iss* < *Iss*.*cSym*, and *Iss* < *Iss*.*cAsym*). Chi-square tests by PAUP based on the nucleotide data sets revealed significant heterogeneity among taxa (*p* < 0.05). To explore the source of base composition heterogeneity within each combined data set, the Chi-square tests were also performed by IQ-TREE. The results showed that several species contributed mainly to the sequence composition heterogeneity, for example, the *Mongoloraphidia harmandi* (Raphidioptera) and the three representatives of Ithonidae (Neuroptera) (Tables A and B in S4 Table). Comparing the number of taxa passing the Chi-square test across data sets indicated that the addition of RNA genes exacerbated the base composition difference among taxa. Removing the third codon positions, translating nucleotides into amino acids and applying alignment masking effectively reduced the composition heterogeneity.

**Table 2:** Saturation test.

Gene regions	NumOTU	*Iss*	*Iss*.*cSym*	P	*Iss*.*cAsym*	P
PCG1	32	0.285	0.808	0.0000	0.554	0.0000
PCG2	32	0.168	0.808	0.0000	0.554	0.0000
PCG3	32	0.666	0.808	0.0000	0.554	0.0000
PCG12	32	0.225	0.814	0.0000	0.570	0.0000
PCG123	32	0.333	0.818	0.0000	0.572	0.0000
rrnS	32	0.336	0.730	0.0000	0.410	0.0000
rrnL	32	0.342	0.766	0.0000	0.475	0.0000
tRNA	32	0.227	0.770	0.0000	0.482	0.0000

For outgroup taxa, two coleopteran species had the higher values of *dN* (avg. 0.2290), *dS* (avg. 5.3490) and *dN/dS* (avg. 0.0428) (Table A in S5 Table). For the ingroup exemplars of Neuropterida, the single representative of Raphidioptera had a similar *dN* (0.2405), *dS* (5.2542) or *dN/dS* (0.0458) value as that of outgroup taxa. Whereas, the remaining ingroups displayed the lower rates of sequence evolution, with the average *dN*, *dS* and *dN/dS* values of 0.1640, 5.0798 and 0.0323 for Megaloptera, and 0.1560, 4.5043 and 0.0357 for Neuroptera. The one-way ANOVA analyses revealed incongruence in the *dN* or *dN/dS* values between groups (*P* < 0.05). However, there was no significant difference in the *dS* values (*P* = 0.4355).

Both the evolutionary rate analysis and the sequence divergence analysis showed the same trend for the protein-coding genes (Table B in S5 Table). The *atp8* and *nad6* genes have undergone accelerated evolution, as evidenced by the highest values of *dN* or *dN/dS* and the highest level of divergence. The *cox1* and *cytb* genes are the relatively conserved protein-coding genes. Alignments based on the secondary structure information reduced the level of divergence of mitochondrial ribosomal RNA genes.

### Secondary structures of mitochondrial rRNA genes

For the newly sequenced neuropteran species, the *rrnS* and *rrnL* were respectively identified between *trnV* (tac) and control region and between *trnL1* (tag) and *trnV* (tac), with length ranging from 778 bp to 799 bp for *rrnS*, and from 1,314 bp to 1,324 bp for *rrnL* (Tables A-D in S3 Table). Both the location and the sequence lengths of *rrnS* and *rrnL* were consistent with other published neuropteran insects.

Fig 1 depicts the inferred secondary structure for the mitochondrial *rrnS* gene of *C*. *nipponensis* EMHAU-15090613, while the remaining *rrnS* secondary structures for ten representatives of Neuropterida are available in Figures A-J in S1 Fig. The alignment derived from these secondary structures spanned 784 nucleotide positions, which contained 402 variable positions.

**Fig 3:**
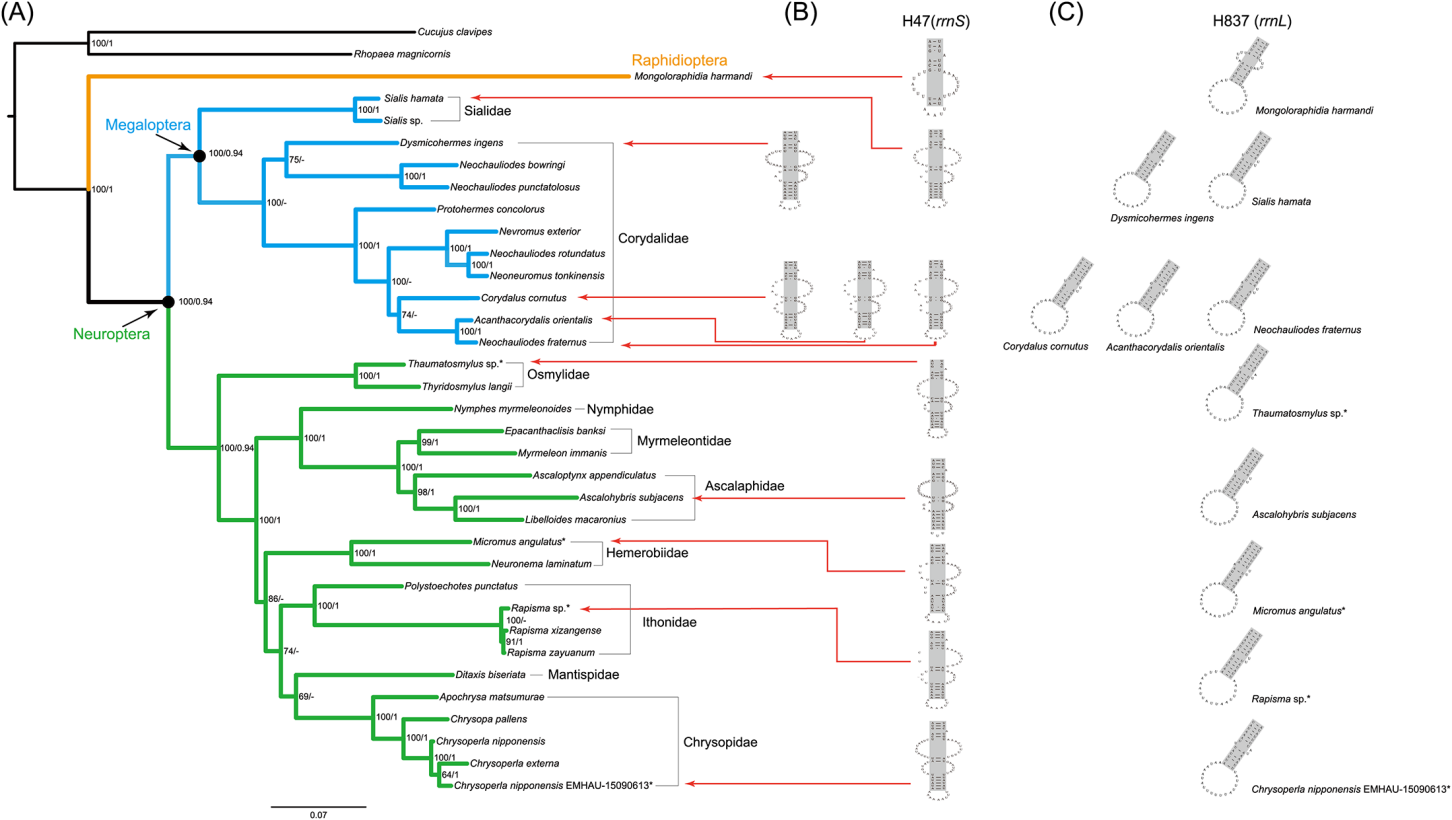
Phylogenetic reconstruction and secondary structure drawings of the helices. (A) Phylogenetic reconstruction from PCG12RNA using software IQ-TREE under the partitioned models automatically selected. Node values represent bootstrap values (left) and posterior probabilities (right). The “-” indicates the relationship not being retrieved by the data set of PCG12RNA using software PhyloBayes under CAT-GTR model. Asterisks designate the species newly sequenced in this study. (B) Secondary structure drawings of the helix 47 in *rrnS* for eleven representative neuropterid species. (C) Secondary structure drawings of the helix 837 in *rrnL* for eleven representative neuropterid species.

**Fig 4:**
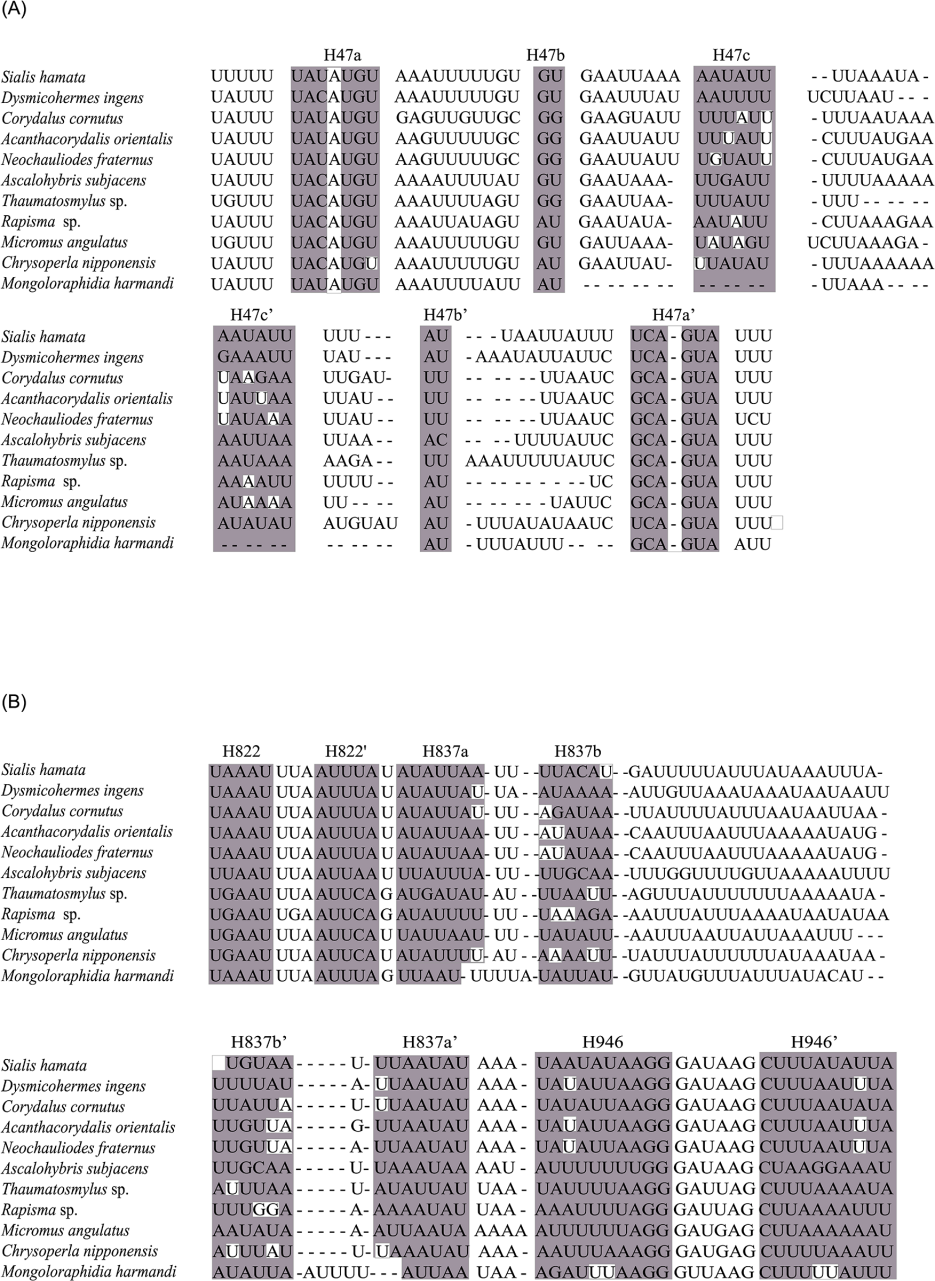
Alignments of helices for eleven representative neuropterid species. (A) Alignments of the helix 47 in *rrnS*. (B) Alignments of the helix 837 in *rrnL*. Helices are highlighted by grey box and numbered as diagrams of the full secondary structures. No color areas in each helix indicate the unpaired regions.

The predicted secondary structures of the mitochondrial *rrnS* genes were similar to those of other arthropods [77,83,84], in which there were four typical domains identified (Fig 1, Figures A-J in S1 Fig and S6 Table). In the Domain I, Megaloptera and Neuroptera shared a similar secondary structure of H47, which was comprised of two long stems, one short stem and three loops (Fig 3B). However, the secondary structure of H47 predicted for *M*. *harmandi* (representing Raphidioptera) contained only one long stem, one short stem and two loops (Fig 3B). An alignment of H47 based on secondary structure information was performed for eleven neuropterid species (Fig 4A). The secondary structure of this motif can serve as a potential synapomorphy supporting the sister-group relationship between Megaloptera and Neuroptera. H17 was another highly conserved motif of Domain I, which included six identical nucleotides across eleven neuropterid *rrnS* sequences. The H885 in the Domain II and the H921, H939 and H1074 in the Domain III were relatively conserved, whose heterogeneity were mainly caused by the *M*. *harmandi*. Domain IV included two helices: H1399 and H1506, which were highly conserved in the sequence composition (conserved positions were 83.33% and 70.00%, respectively) (S6 Table). In particular, all neuropterids had an identical nucleotide composition for the H1506, with the exception of *M*. *harmandi*. Domain III and Domain IV were more conserved than Domain I and Domain II, with regard to the secondary structure and the base composition.

Secondary structure diagrams for the mitochondrial *rrnL* gene of *C*. *nipponensis* EMHAU-15090613 is shown in Fig 2, while the remaining *rrnL* secondary structures for ten representatives of Neuropterida are available in Figures A-J in S2 Fig. All predicted secondary structures of *rrnL* comprised of five canonical structural domains (I-II, IV-VI) and 44 helices (Fig 2, Figures A-J in S2 Fig and S7 Table), which were also found in other arthropod taxa [77,83–86]. The inferred *rrnL* secondary structures did not contain domain III, due to a shorten sequence region between domain II and IV (Fig 2 and Figures A-J in S2 Fig). The alignment of *rrnL* genes for eleven neuropterids based on the secondary structures spanned 1,250 nucleotide positions, with 679 variable positions. Domains I-II were more variable than the rest of domains (S7 Table). The secondary structures of H837 predicted for Megaloptera and Neuroptera were similar (Fig 3C). Whereas, the *M*. *harmandi* had a distinct structure of this helix, which contained an obviously longer unpaired region in the middle region (Fig 3C). Fig 4B illustrated the alignment derived from the secondary structure of H837 for eleven neuropterid species. The structure information of this helix may also be potential for the phylogenetic inference. Domains IV-VI were highly conserved, in which H1755, H1835, H1906, H1925, H2064, H2359, H2507 and H2735 had more than 75% sequence similarity, respectively.

### Phylogenetic analyses

#### Phylogeny of Neuropterida

For the inter-order relationships within Neuropterida, the hypothesis of (Raphidioptera + (Megaloptera + Neuroptera)) was consistently recovered by all combined analyses (e.g. Fig 3A), albeit with strong supports for main nodes (BP > 90, PP > 0.90).

Across ML analyses, the differences occurred in the resolution of internal relationships within the Megaloptera and those within the Neuroptera. In the Megaloptera, two families were represented by more than two exemplars, namely the Sialidae and the Corydalidae. The monophyly of both families were strongly supported (BP = 100). At the genus level, the *Neochauliodes* was retrieved as non-monophyletic. Four species of *Neochauliodes* scattered among three clades in all ML analyses. Our sampling within the most diverse Neuroptera included 20 species representing eight families. Four families with more than one exemplars were congruently recovered as monophyletic: Osmylidae, Ithonidae, Hemerobiidae, and Chrysopidae. However, the Myrmeleontidae was retrieved as non-monophyletic with respect to Ascalaphidae in the ML analyses based on Aliscore_PCG and Aliscore_PCG-SS. All other ML analyses supported the Myrmeleontidae as a monophyletic group. In addition, the Ascalaphidae was recovered as monophyletic by several data sets (i.e. PCG, PCGRNA, PCG12RNA, Aliscore_PCG, Aliscore_PCGRNA and Aliscore_PCG12RNA). The remaining ML analyses recovered the Ascalaphidae to be a paraphyletic group, with *Ascalohybris subjacens* splitting as an independent branch. For the inter-familial relationships within Neuroptera, the Osmylidae was recovered as the most basal lineage. All other neuropteran taxa could be divided into two large groups. One included three families: Nymphidae, Myrmeleontidae, and Ascalaphidae. The other contained the Ithonidae, Mantispidae, Hemerobiidae, and Chrysopidae. The interrelationships within each grouping varied with different data sets. However, two sister-group relationships were often recovered: (Myrmeleontidae + Ascalaphidae) and (Hemerobiidae + Chrysopidae).

Bayesian analyses under the site-heterogeneous model produced the similar tree topologies to ML analyses. The monophyly of Neuropterida was strongly supported, and the sister-group relationship (Megaloptera + Neuroptera) was consistently recovered. Furthermore, the branching patterns within the Megaloptera and the Neuroptera were basically identical to those in ML trees. Bayesian and ML analyses differed only in the resolution of deep nodes. In the Bayesian analyses of Aliscore_PCGRNA, Aliscore_PCG12RNA, Aliscore_PCGRNA-SS and Aliscore_PCG12RNA-SS, the relationship among three neuropteridan orders was almost unresolved owing to the extremely short internal branch. At the family level, Bayesian analysis from Aliscore-PCG recovered a sister-group relationship between Hemerobiidae and Chrysopidae, while ML analysis from the same data set did not.

#### Effect of data treatment methods

Comparison between topologies from various data treatment methods showed that removing the third codon positions and translating nucleotides into amino acids had a marked influence on the ingroup relationships. In the analyses of PCG12 and PCG_AA, the monophyly of Ascalaphidae was not retrieved, while the sister group (Hemerobiidae + Chrysopidae) was always recovered. In contrast, sequence alignment masking (e.g. Aliscore_PCG) did not significantly alter the ingroup relationships under the same tree reconstruction method.

Realignment of mitochondrial *rrnS* gene sequences based on the secondary structure information significantly improved the resolution of relationships within Neuropterida (S3 Fig). However, the alignment of *rrnL* derived from the secondary structure produced an unexpected topology, in which the *M*. *harmandi* nested within the Megaloptera and was recovered as sister to the Sialidae. This may be due to the more hyper-variable regions contained in *rrnL* gene sequences, for which they are difficult to be aligned unambiguously even with reference to secondary structure information.

## Discussion

### NGS approach to mitogenome assembly

Mitogenome sequences have proved to be useful for studying Neuropterida phylogeny [6,18,25,28,30,87]. However, the availability of complete mitogenomes for neuropteridan insects is still limited. To date, there is no report on the NGS technology applied to the determination of neuropterid mitogenome. In this study, we employed high-throughput pooled sequencing approach to mitogenome assembling, and successfully reconstructed four complete mitogenomes of Neuroptera. Combined with the existing neuropteran mitogenome sequences, the newly sequenced mitogenomes were utilized for phylogenetic reconstruction of Neuropterida. The tree building results presented congruent phylogenetic hypotheses with recent studies on the higher-level phylogeny of Neuropteria [7,16,20,23,28,29,30], in which all four newly determined mitogenome sequences were consistently positioned in the clade including their close relatives. In addition, the gene arrangement, nucleotide composition, gene length, anti-codons of *tRNA*, start and stop codon usage of protein-coding genes and sequence evolutionary rate were similar to most of neuropteran mitogenomes published. All these results validated the applicability of acquiring large number of neuropterid mitogenomes from de novo assembly of high-throughput pooled sequencing. Furthermore, more insect species can be added into the pool to improve sequencing effectiveness from the point of view of deeper sequencing coverage applied. At present, sequencing cost has been decreased to < US$300 for constructing a single library and generating 10 G data by a standard NGS platform. Thus, the NGS approach for reconstructing mitogenomes will become popular for insect mitochondrial phylogenomics due to its relatively easy protocol and remarkably lower cost.

### Genome organization

Just when we finished writing this paper, another research of mitogenomic phylogeny of Neuropterida had been published [30]. In the analysis of Wang et al. (2017) [30], a greater number of mitogenomes of represented Neuropterida were sequenced by PCR amplification and traditional Sanger sequencing method. Wang et al. (2017) [30] revealed the *tRNA* arrangement of *trnW*(tca)-*trnC*(gca)-*trnY*(gta) (i.e. WCY) as an ancestral gene organization harbored by the neuropteran families Coniopterygidae, Nevrorthidae, Osmylidae and Sisyridae. Furthermore, they suggested the *tRNA* arrangement of WCY as the potential evidence for the basal position of Osmylidae in Neuroptera. However, our sequencing result demonstrated that the *tRNA* arrangement of *trnC*(gca)-*trnW*(tca)-*trnY*(gta) (i.e. CWY in *Thaumatosmylus* sp.) also occurred in the Osmylidae. Therefore, whether the arrangement of this *tRNA* cluster can be regarded as an evidence for neuropterid phylogeny requires additional data.

### Comparative analysis of mitochondrial *rRNA* secondary structures

By comparing mitochondrial *rRNA* secondary structures between species, conserved motifs and highly variable portions within each canonical structural domain were identified (Figs 1 and 2, Figures A-J in S1 Fig, Figures A-J in S2 Fig, and S6 and S7 Tables). Subsequently, the conserved sequence motifs and their associated secondary structure elements were utilized to identify the homologous positions in sequences. In this paper, we analyzed the complete mitochondrial *rrnS* and *rrnL* gene sequences for eleven neuropterid species. The results demonstrated that realigned *rRNA* data could improve the resolution of relationships within Neuropterida (S3 Fig). Two conserved motifs (i.e. H47 for *rrnS* and H837 for *rrnL*) were found to be the potential evidence for a close relation between Megaloptera and Neuroptera (Fig 3B and 3C). In the prior studies, only one neuropteran insect mitochondrial *rrnS* and *rrnL* secondary structures were predicted, namely the *Libelloides macaronius* [87]. Some regions inferred for the secondary structures of *L*. *macaronius* mitochondrial *rrnS* and *rrnL* were different from other insects published [77,78]. For example, the Domain I proposed for the secondary structure of *L*. *macaronius* mitochondrial *rrnL* was distinct from the commonly utilized model of *A*. *mellifera* [77]. The prediction for the structure of this region by Negrisolo et al. (2011) [87] was not corroborated by the current study, either. The eleven neuropterid *rrnL* sequences can be inferred as the *A*. *mellifera* model [77]. With a denser taxon sampling, the diagrams of mitochondrial *rrnS* and *rrnL* secondary structures presented in this study can be useful to improve the accuracy of the neuropterid secondary structures, and allow better visualization and comparison of mitochondrial *rRNA* structural features. These will be of fundamental importance to their application in phylogenetic studies and to understanding the function of these molecules for neuropterid insects.

### Neuropterida phylogeny

With respect to the higher-level phylogeny of Neuropterida, the sister-group relationship between Raphidioptera and Megaloptera has long been recognized [8,9,88] by some of Neuropterida systematists. This view was also advocated in the study of Beutel et al. (2010) [21]. Synapomorphies supporting this relation were mainly from the morphological structures of larval and adult head. But these characters were re-considered as symplesiomorphic by Aspöck and Haring (2012) [19]. In the trees recovered in this study, only two analyses based on the single *rRNA* gene alignments (i.e. *rrnL*-SS and *rrnS*) under ML inference recovered a close relationship between Raphidioptera and Megaloptera, but with weak nodal support values (BP < 75). This result was likely to be an artifact of inaccurate alignments of mitochondrial *rRNA* genes. Because the *rrnL* gene sequences contained more highly variable regions, while the automatically aligned *rrnS* gene sequences had insufficient phylogenetic information on the resolution of deep nodes in the neuropterid tree. In contrast, utilizing secondary structure information resulted in a marked increase in the phylogenetic signal and the lower sequence divergence of alignment of *rrnS*-SS. The conserved sites, variable sites and parsimony-information sites relative to all sites for *rrnS*-SS and *rrnS* are 254/548/433/802 and 282/492/401/774, respectively. Thus, a congruent topology with the well-supported tree from the combined data can be recovered by *rrnS*-SS alignment.

The majority of trees from mitogenomic data provided strong support for the hypothesis of (Raphidioptera + (Megaloptera + Neuroptera)). In addition, we provided a potential evidence from the secondary structures of two conserved motifs (i.e. H47 in *rrnS* and H837 in *rrnL*) for this inter-ordinal relationship. This branching pattern was also supported by recent morphological [23,29] and molecular [7,16,28,30] studies. Supporting evidence mainly included the larval morphological characters [23], the wing base structures [29] and the mitogenomic data [30]. In the study of Wang et al. (2017) [30], significant compositional heterogeneity of mitogenome sequences across lineages had an adverse effect on the resolution of higher-level phylogeny of Neuropterida, especially under homogeneous model and parsimony analyses. More recently, Winterton et al. (2017) [20] provided the most comprehensive study of Neuropterida phylogeny using anchored phylogenomics to date. And they recovered the monophyletic Raphidioptera as sister to the rest of Neuropterida in all ML and Bayesian analyses, with strong statistical support [20]. However, they indicated that sequence heterogeneity had no apparent negative impact on phylogeny estimation. Our sequence characteristic analysis also showed a rate heterogeneity across miogenome sequences of three neuropterid orders. The monophyly of each order and the similar inter-ordinal relationships as recent studies [20,30] were retrieved in all analyses, regardless of models or algorithms utilized. The difference at the lower-level relationships between studies may be due to the various taxon sampling. Because the effect of sequence heterogeneity is likely to be exacerbated with expanding sequence data from limited species coverage. We should acknowledge that all current studies included still sparse taxon sampling for a highly diverse Neuropterida, which might have an influence on the resulting phylogeny. Addition of more mitogenome or the whole genome-wide data, especially those for species of Raphidioptera, to future neuropteridan phylogenetic studies can provide new insights into the evolutionary relationships among three orders. Thus, we considered the current phylogenetic result on the ordinal relationships of Neuropterida to be still tentative.

The monophyly of Megaloptera was questioned by several previous studies [19,20]. However, the results of these studies were considered as incredible by Aspöck and Haring (2012) [19], due to the various tree searching algorithms, character or taxon sampling limitation. In this paper, the sister-group relationship between Sialidae and Corydalidae were consistently recovered by the present mitogenomic data, with strong nodal support (BP = 100, PP = 1). This relationship was also supported by recent morphological [5,89] and molecular studies [4,30]. Therefore, our results confirmed a monophyletic Megaloptera, encompassing two families of Sialidae and Corydalidae.

Within Neuroptera, eight families were included in the present study. The Osmylidae were recovered with strong support (BP = 100, PP = 1) as the earliest offshoot to all other neuropteran families. The basal position of Osmylidae was largely congruent with previous molecular study based on the single nuclear and mitochondrial gene fragments [4] and the mitogenomic data [30]. The clade (Nymphidae + (Myrmeleontidae + Ascalaphidae)) was recovered by most of combined analyses. The same result was also retrieved by some prior studies [4,5,19,22,23,90,91,92,93]. However, Michel et al (2017) [94] showed that Nemopteridae had a closer relationship to the clade (Myrmeleontidae + Ascalaphidae) than Nymphidae. Winterton et al. (2017) [20] also placed the Nemopteridae as sister to the assemblage including Myrmeleontidae and Ascalaphidae. Therefore, the clade comprising (Nymphidae + (Myrmeleontidae + Ascalaphidae)) recovered by the present data may be due to the absence of Nemopteridae and Psychopsidae. More extensive sampling is needed to definitively elucidate relationships between these families. In the study by Winterton et al. (2017) [20], the nested position of Ascalaphidae led to a paraphyletic Myrmeleontidae. Similarly, the paraphyly of Myrmeleontidae was also proposed by Wang et al. (2017) [30]. However, most of our analyses retrieved the Ascalaphidae and Myrmeleontidae as diverging clades. The cluster of the remaining four families recovered in our analyses had the same constitution as those in the study by Haring and Aspöck (2004) [4], who recognized the group (Ithonidae + Polystoechotidae + Hemerobiidae + Chrysopidae + Mantispidae) according to A + T content of nuclear *18S rRNA* sequences. Of which, the Polystoechotidae is more often considered as a member of the family Ithonidae [20,30]. Winterton and Makarkin (2010) [95] actually demonstrated the clade comprising Ithonidae and Polystoechotidae as a monophyletic group (i.e. an unique family) based on molecular, morphological and fossil evidence. In this study, we argued for merging the Polystoechotidae into the family Ithonidae. The limited taxon sampling led to an aberrant hierarchy arrangement of four families in several analyses (e.g. the topology depicted in Fig 3A). This branch pattern is different from the most recent analyses. In particular, the Chrysopidae was retrieved in a terminal position, which contrasted with a deeper placement within Neuroptera in two recent studies [20,30]. Nevertheless, the sister-group (Hemerobiidae + Chrysopidae) was frequently recovered in our analyses (e.g. the ML and Bayesian analyses of PCG, PCG12 and PCG_AA). This result agreed with the previous molecular studies by Haring and Aspöck (2004) [4] and Wang et al. (2017) [30], but disagreed with that of Winterton et al. (2017) [20].

## Supporting information

S1 Fig Secondary structure drawing of the complete mitochondrial *rrnS* gene for ten neuropterid species. (A) *Acanthacorydalis orientalis*, (B) *Ascalohybris subjacens*, (C) *Corydalus cornutus*, (D) *Dysmicohermes ingens*, (E) *Micromus angulatus*, (F) *Mongoloraphidia harmandi*, (G) *Neochauliodes fraternus*, (H) *Thaumatosmylus* sp., (I) *Rapisma* sp., and (J) *Sialis hamate*.
(ZIP)

S2 Fig Secondary structure drawing of the complete mitochondrial *rrnL* gene for ten neuropterid species. (A) *Acanthacorydalis orientalis*, (B) *Ascalohybris subjacens*, (C) *Corydalus cornutus*, (D) *Dysmicohermes ingens*, (E) *Micromus angulatus*, (F) *Mongoloraphidia harmandi*, (G) *Neochauliodes fraternus*, (H) *Thaumatosmylus* sp., (I) *Rapisma* sp., and (J) *Sialis hamate*.
(RAR)

S3 Fig Phylogenetic reconstruction from the data set of *rrnS-*SS using software IQ-TREE under GTR+G+I model. Node values represent bootstrap values.
(TIF)

S1 Table Taxa used in this study. (XLSX)

S2 Table The partition schemes and best-fitting models selected by PartitionFinder for each dataset. (XLSX)

S3 Table The organization of four newly sequenced mitogenomes. (XLSX)

S4 Table The Chi-square tests. (XLSX)

S5 Table The evolutionary rate analyses conducted for each species and for each gene partition. (XLSX)

S6 Table The nucleotide composition in each helix of the secondary structures of *rrnS* in 11 Neuropterida mitogenomes. (XLSX)

S7 Table The nucleotide composition in each helix of the secondary structures of *rrnL* in 11 Neuropterida mitogenomes. (XLSX)

S1 File All sequence alignment files in PHYLIP format compiled in this study. (ZIP)

S2 File All ML trees in NEXUS format reconstructed in this study. (ZIP)

S3 File All Bayesian trees in NEXUS format reconstructed in this study. (ZIP)
