# Supplementary figures and images for: Insight into higher-level phylogeny of Neuropterida: Evidence from secondary structures of mitochondrial rRNA genes and mitogenomic data
Source: PLoS One. 2018 Jan 30;13(1):e0191826. doi: 10.1371/journal.pone.0191826 (PMC5790268; doi:10.1371/journal.pone.0191826)

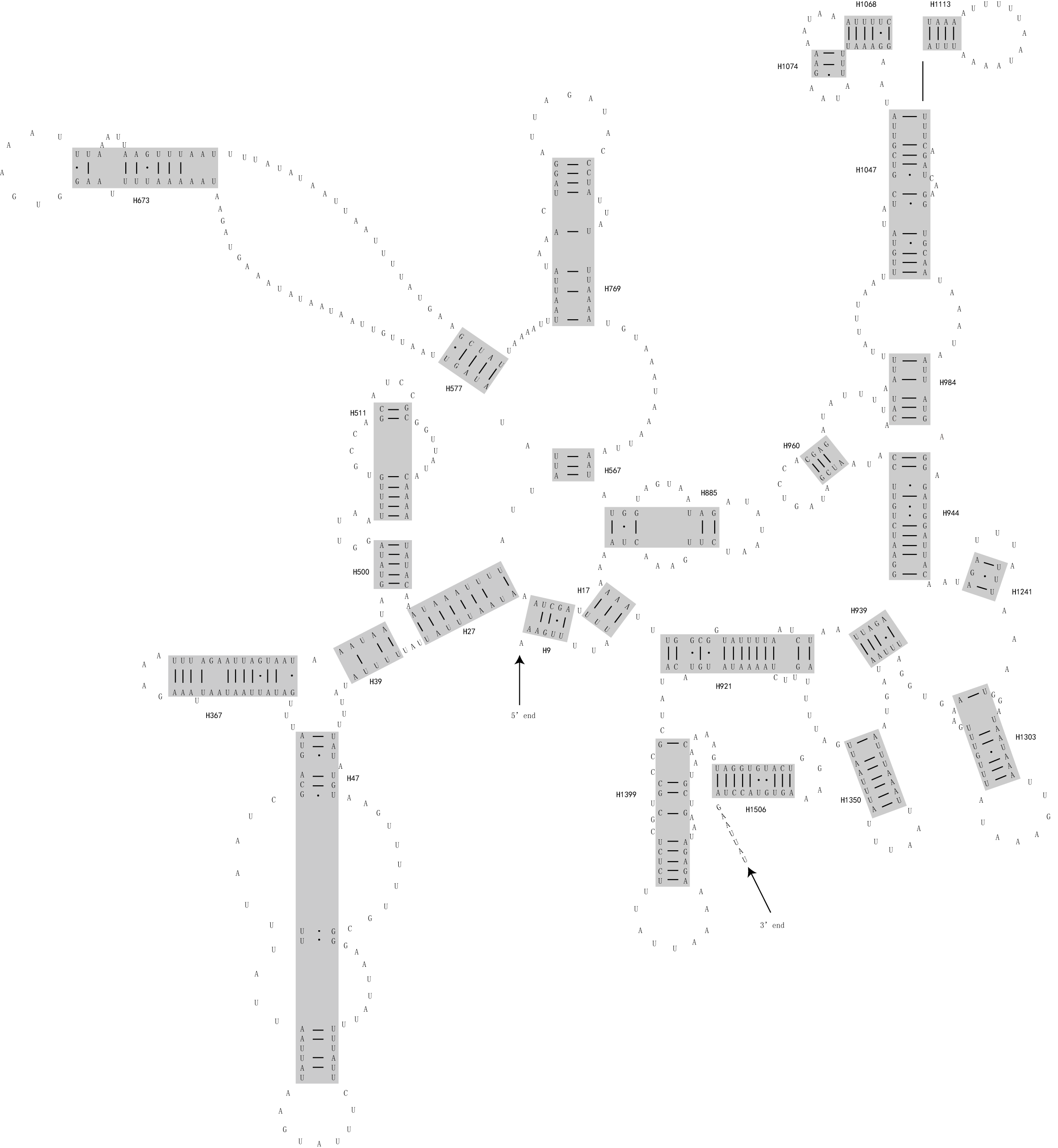

Supplement: S1 Fig — (A) Acanthacorydalis orientalis, (B) Ascalohybris subjacens, (C) Corydalus cornutus, (D) Dysmicohermes ingens, (E) Micromus angulatus, (F) Mongoloraphidia harmandi, (G) Neochauliodes fraternus, (H) Thaumatosmylus sp., (I) Rapisma sp., and (J) Sialis hamate. (ZIP) [file pone.0191826.s001.zip › Fig S1A.tif]

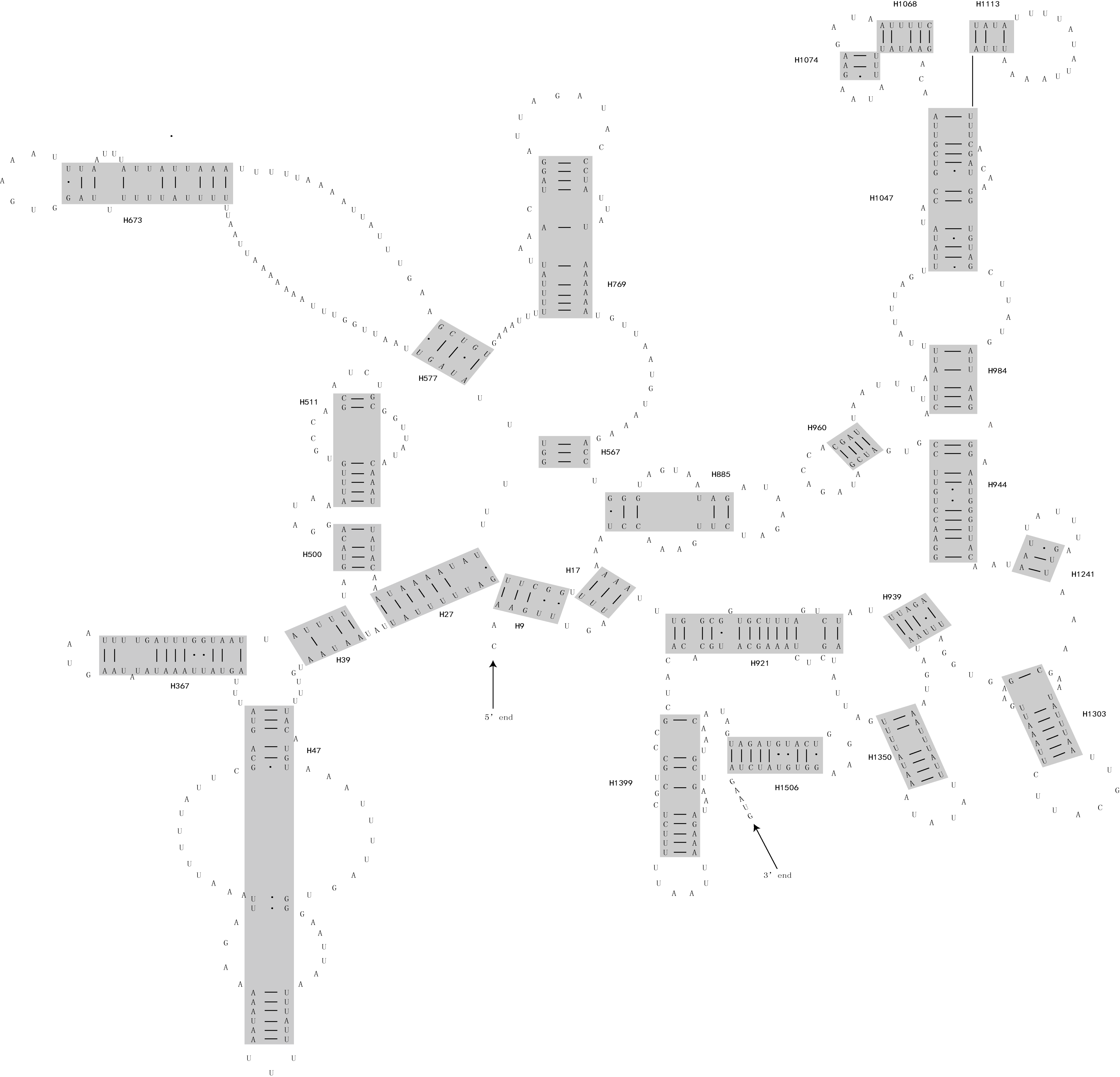

Supplement: S1 Fig — (A) Acanthacorydalis orientalis, (B) Ascalohybris subjacens, (C) Corydalus cornutus, (D) Dysmicohermes ingens, (E) Micromus angulatus, (F) Mongoloraphidia harmandi, (G) Neochauliodes fraternus, (H) Thaumatosmylus sp., (I) Rapisma sp., and (J) Sialis hamate. (ZIP) [file pone.0191826.s001.zip › Fig S1B.tif]

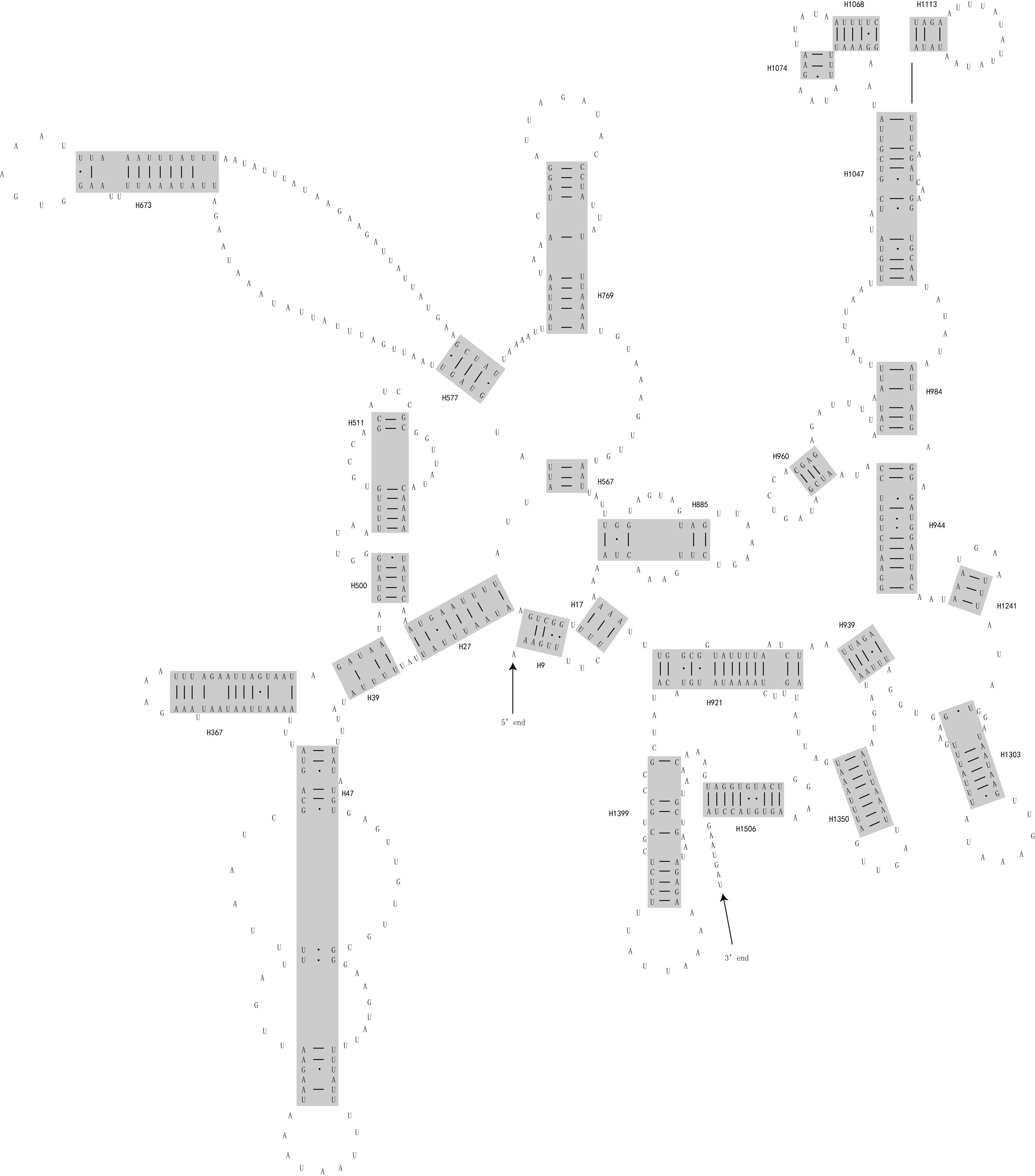

Supplement: S1 Fig — (A) Acanthacorydalis orientalis, (B) Ascalohybris subjacens, (C) Corydalus cornutus, (D) Dysmicohermes ingens, (E) Micromus angulatus, (F) Mongoloraphidia harmandi, (G) Neochauliodes fraternus, (H) Thaumatosmylus sp., (I) Rapisma sp., and (J) Sialis hamate. (ZIP) [file pone.0191826.s001.zip › Fig S1C.tif]

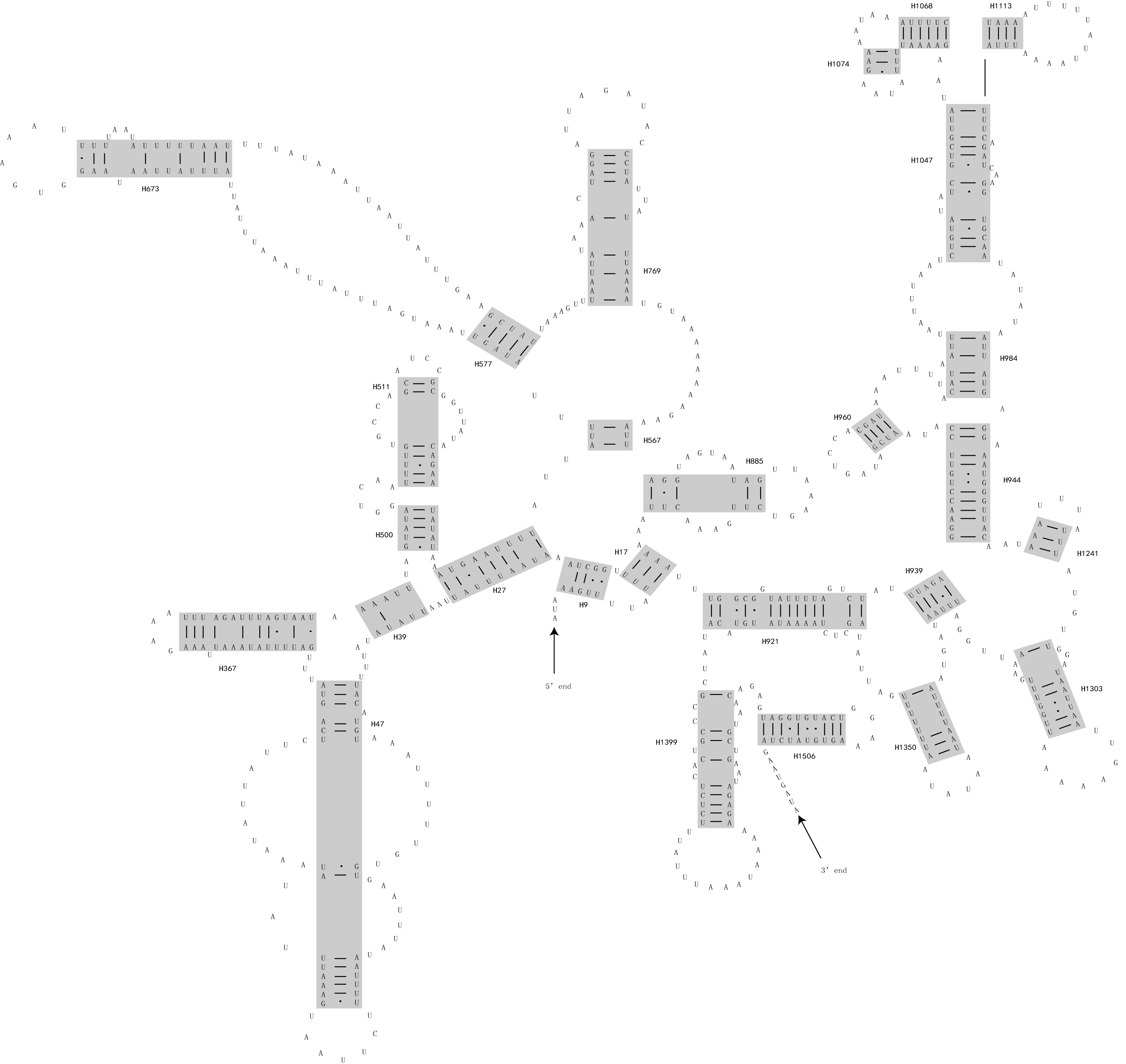

Supplement: S1 Fig — (A) Acanthacorydalis orientalis, (B) Ascalohybris subjacens, (C) Corydalus cornutus, (D) Dysmicohermes ingens, (E) Micromus angulatus, (F) Mongoloraphidia harmandi, (G) Neochauliodes fraternus, (H) Thaumatosmylus sp., (I) Rapisma sp., and (J) Sialis hamate. (ZIP) [file pone.0191826.s001.zip › Fig S1D.tif]

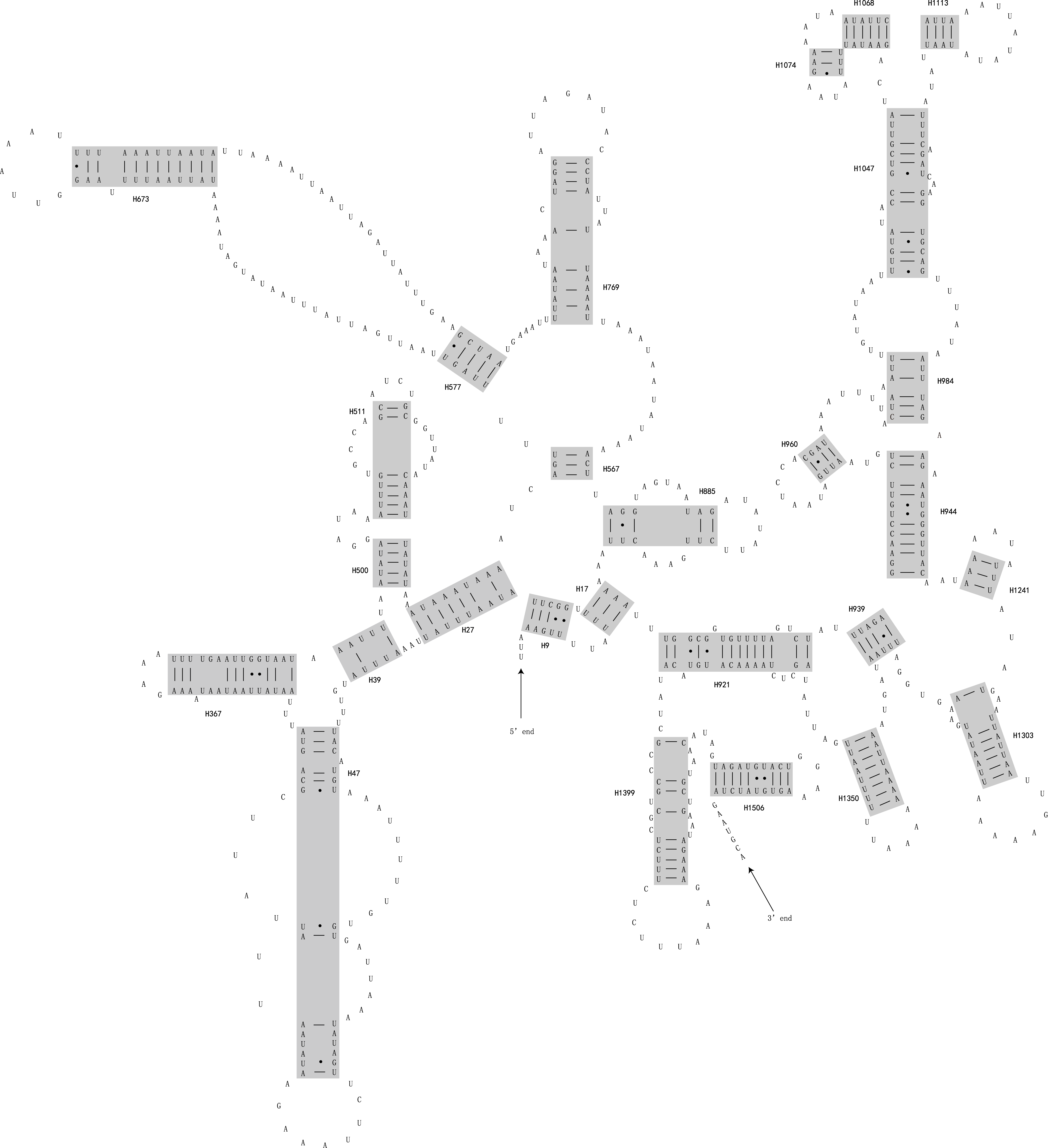

Supplement: S1 Fig — (A) Acanthacorydalis orientalis, (B) Ascalohybris subjacens, (C) Corydalus cornutus, (D) Dysmicohermes ingens, (E) Micromus angulatus, (F) Mongoloraphidia harmandi, (G) Neochauliodes fraternus, (H) Thaumatosmylus sp., (I) Rapisma sp., and (J) Sialis hamate. (ZIP) [file pone.0191826.s001.zip › Fig S1E.tif]

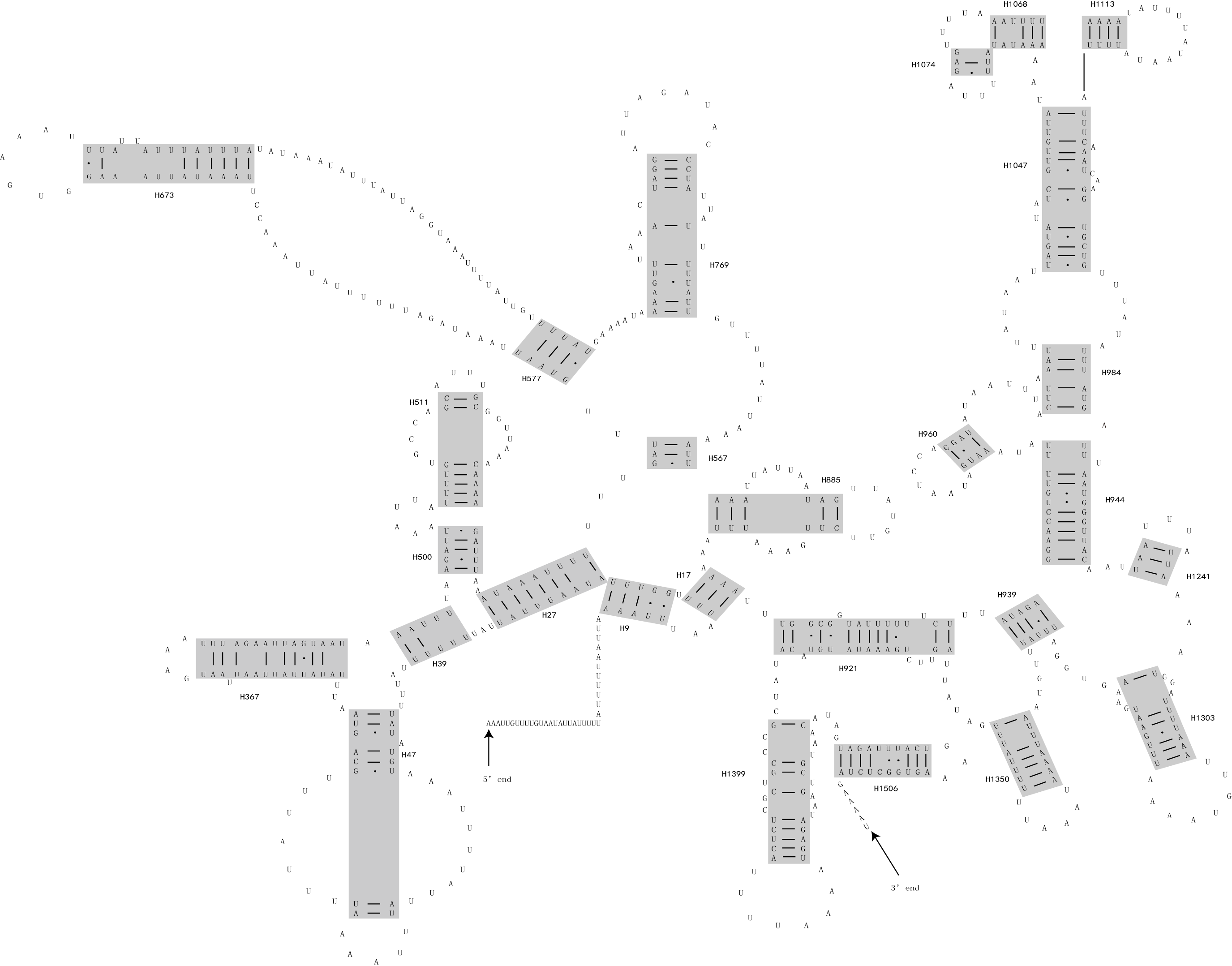

Supplement: S1 Fig — (A) Acanthacorydalis orientalis, (B) Ascalohybris subjacens, (C) Corydalus cornutus, (D) Dysmicohermes ingens, (E) Micromus angulatus, (F) Mongoloraphidia harmandi, (G) Neochauliodes fraternus, (H) Thaumatosmylus sp., (I) Rapisma sp., and (J) Sialis hamate. (ZIP) [file pone.0191826.s001.zip › Fig S1F.tif]

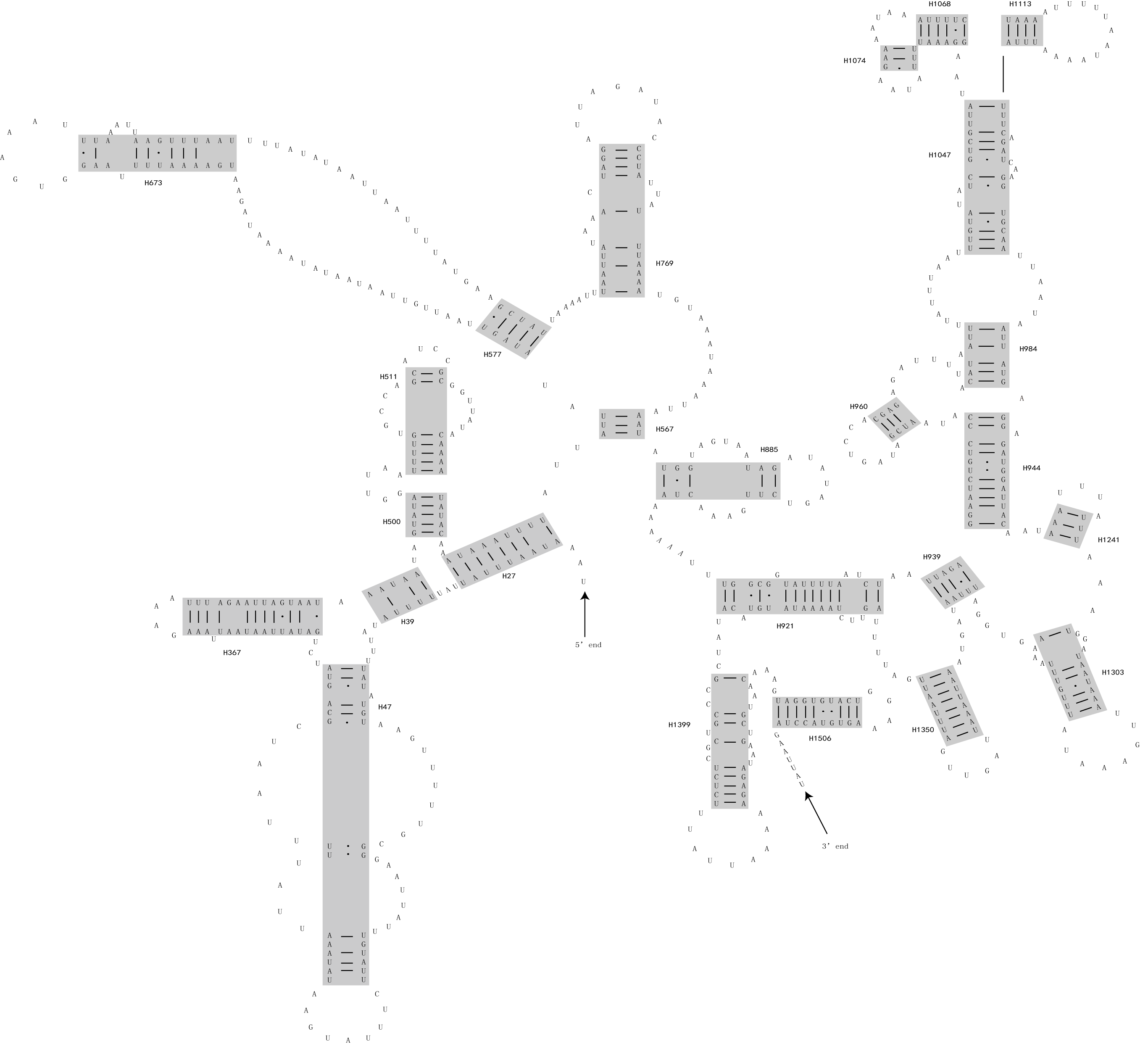

Supplement: S1 Fig — (A) Acanthacorydalis orientalis, (B) Ascalohybris subjacens, (C) Corydalus cornutus, (D) Dysmicohermes ingens, (E) Micromus angulatus, (F) Mongoloraphidia harmandi, (G) Neochauliodes fraternus, (H) Thaumatosmylus sp., (I) Rapisma sp., and (J) Sialis hamate. (ZIP) [file pone.0191826.s001.zip › Fig S1G.tif]

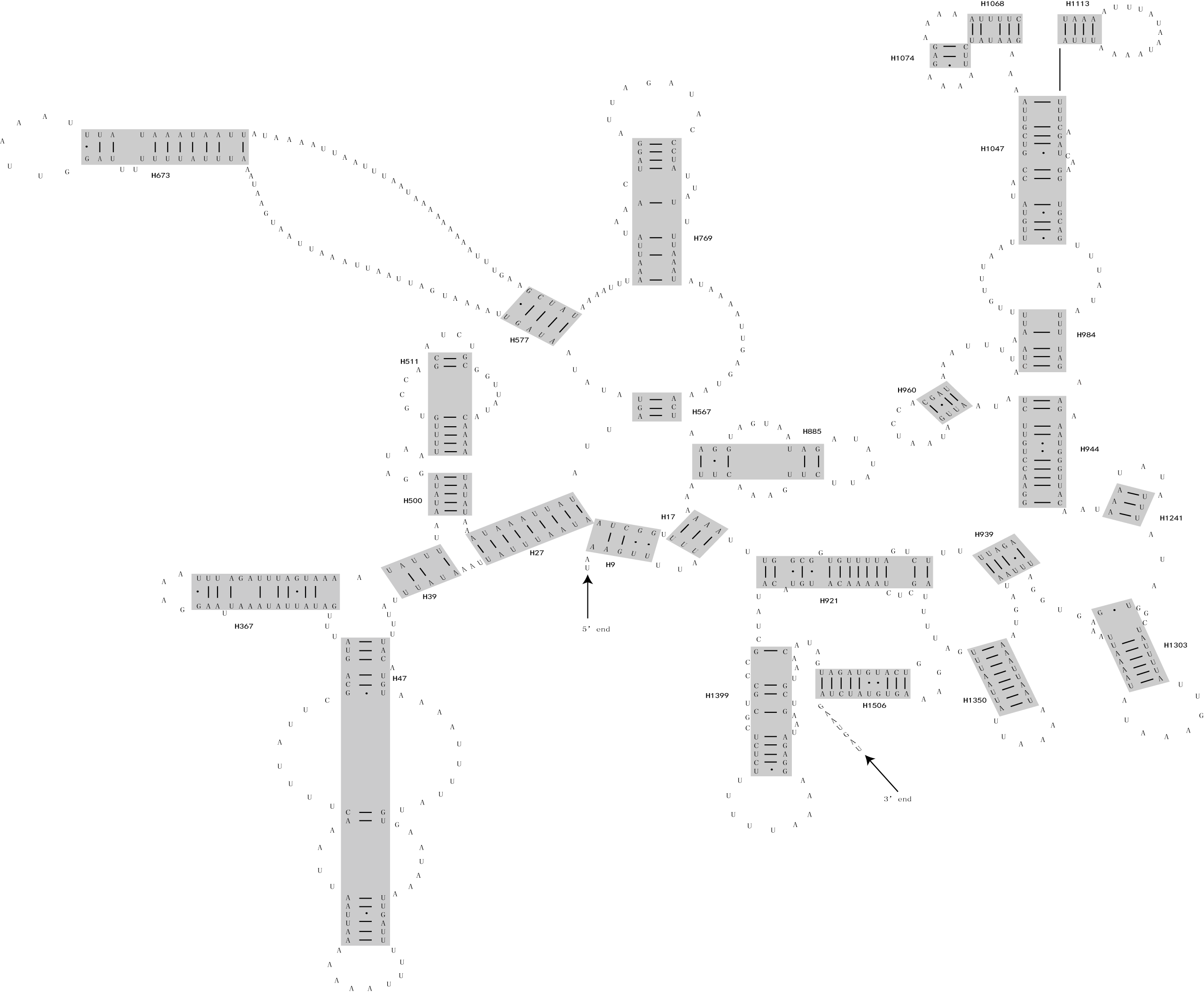

Supplement: S1 Fig — (A) Acanthacorydalis orientalis, (B) Ascalohybris subjacens, (C) Corydalus cornutus, (D) Dysmicohermes ingens, (E) Micromus angulatus, (F) Mongoloraphidia harmandi, (G) Neochauliodes fraternus, (H) Thaumatosmylus sp., (I) Rapisma sp., and (J) Sialis hamate. (ZIP) [file pone.0191826.s001.zip › Fig S1H.tif]

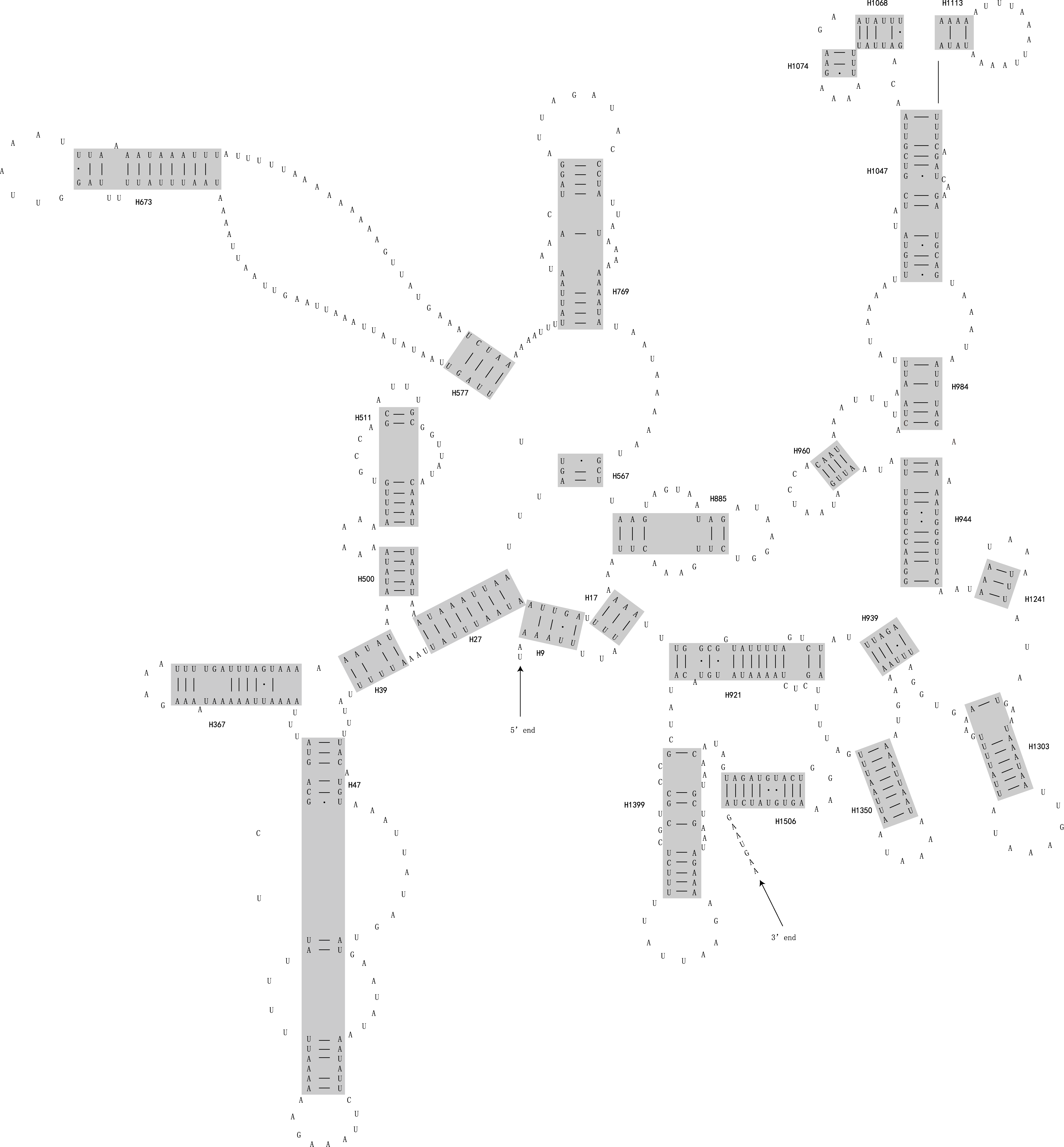

Supplement: S1 Fig — (A) Acanthacorydalis orientalis, (B) Ascalohybris subjacens, (C) Corydalus cornutus, (D) Dysmicohermes ingens, (E) Micromus angulatus, (F) Mongoloraphidia harmandi, (G) Neochauliodes fraternus, (H) Thaumatosmylus sp., (I) Rapisma sp., and (J) Sialis hamate. (ZIP) [file pone.0191826.s001.zip › Fig S1I.tif]

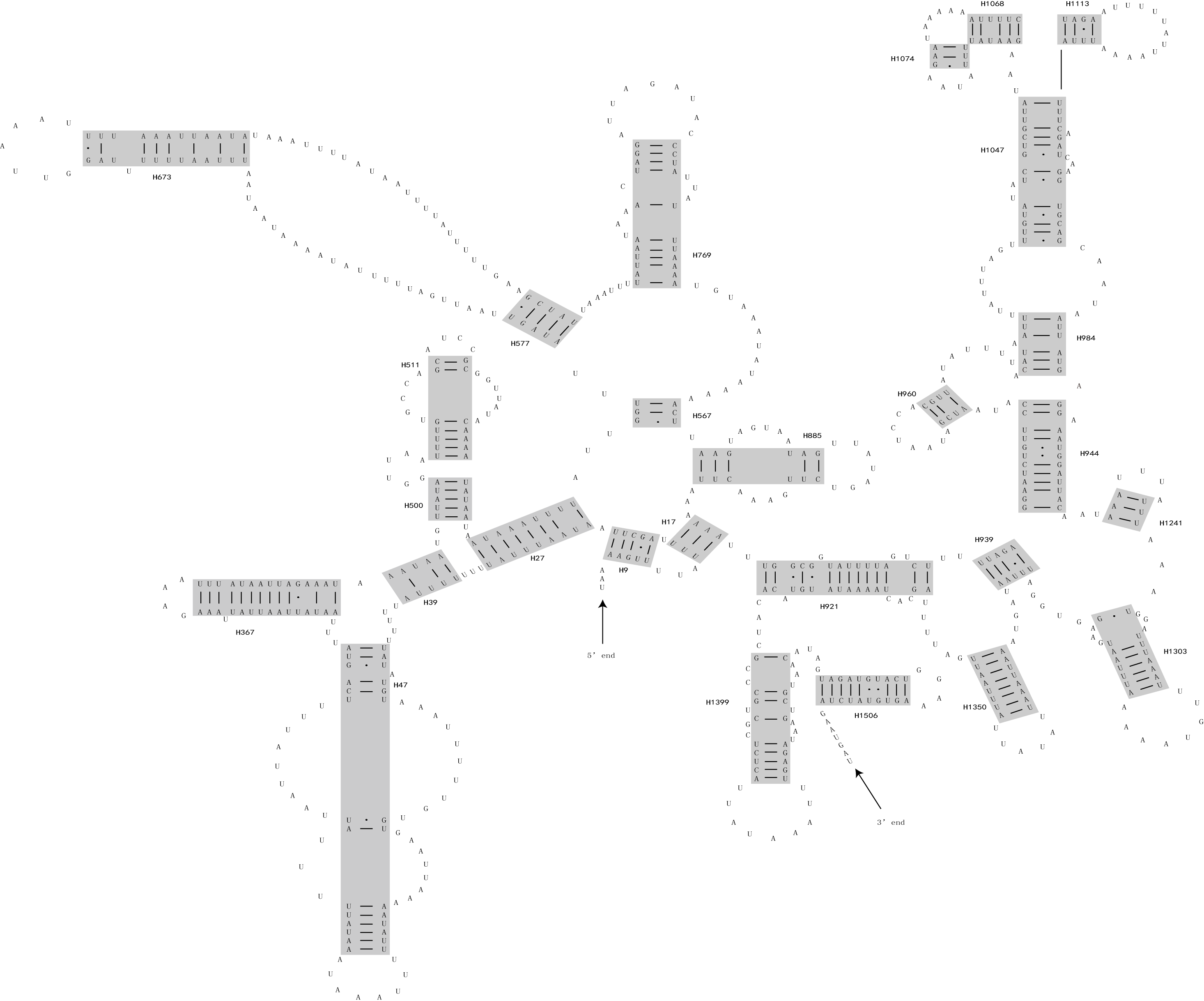

Supplement: S1 Fig — (A) Acanthacorydalis orientalis, (B) Ascalohybris subjacens, (C) Corydalus cornutus, (D) Dysmicohermes ingens, (E) Micromus angulatus, (F) Mongoloraphidia harmandi, (G) Neochauliodes fraternus, (H) Thaumatosmylus sp., (I) Rapisma sp., and (J) Sialis hamate. (ZIP) [file pone.0191826.s001.zip › Fig S1J.tif]

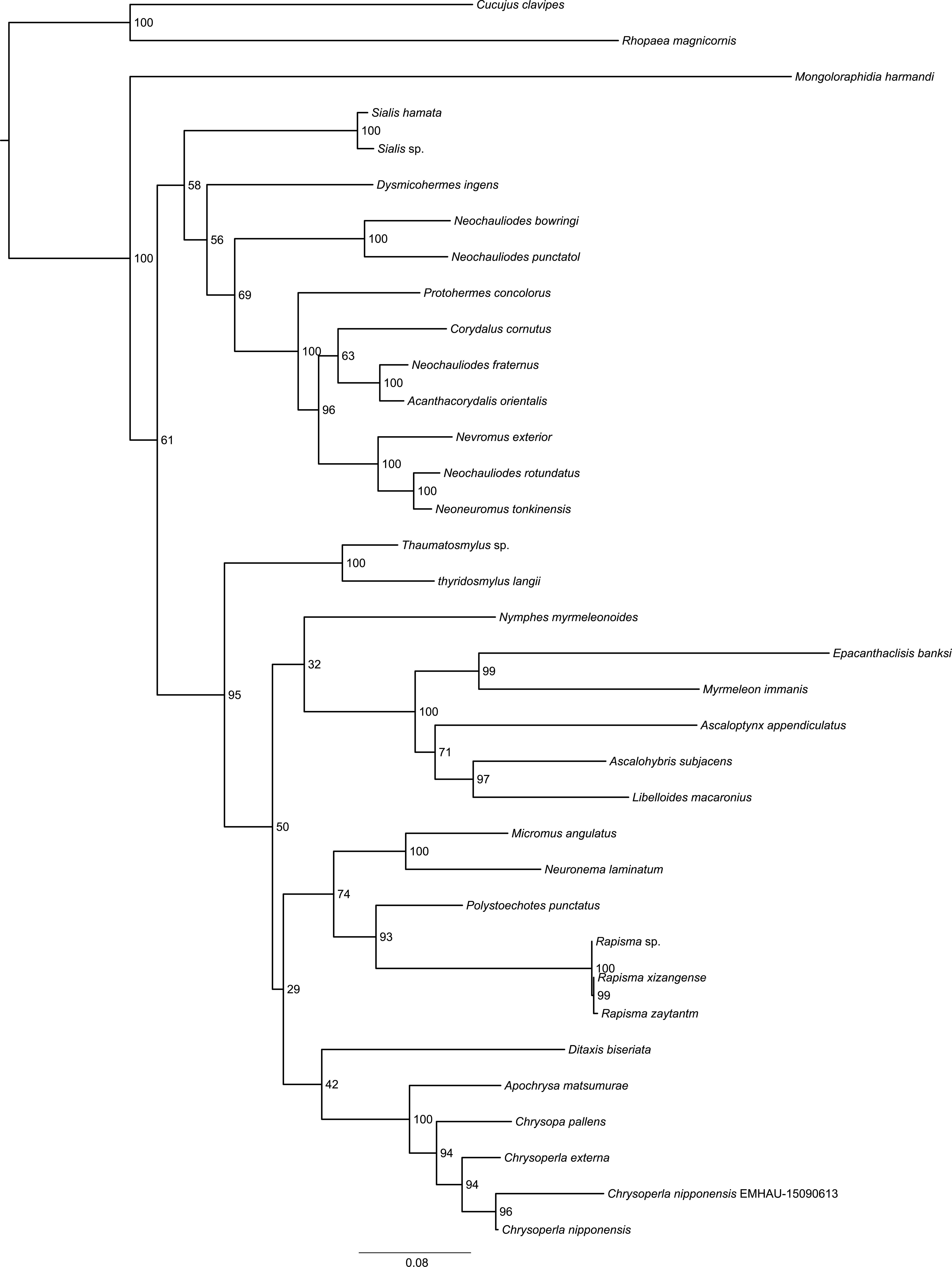

Supplement: S3 Fig — Node values represent bootstrap values. (TIF) [file pone.0191826.s003.tif]
